# In Vitro Evaluation of Self-Nano-Emulsifying Drug Delivery Systems (SNEDDS) Containing Room Temperature Ionic Liquids (RTILs) for the Oral Delivery of Amphotericin B

**DOI:** 10.3390/pharmaceutics12080699

**Published:** 2020-07-25

**Authors:** Eleni Kontogiannidou, Thomas Meikopoulos, Helen Gika, Emmanuel Panteris, Ioannis S. Vizirianakis, Anette Müllertz, Dimitrios G. Fatouros

**Affiliations:** 1 Laboratory of Pharmaceutical Technology, Department of Pharmaceutical Sciences, Aristotle University of Thessaloniki, GR-54124 Thessaloniki, Greece; ekontogi@pharm.auth.gr; 2 Laboratory of Analytical Chemistry, Department of Chemistry, Aristotle University of Thessaloniki, GR-54124 Thessaloniki, Greece; meikthom@chem.auth.gr; 3 Laboratory of Forensic Medicine and Toxicology, School of Medicine, Aristotle University of Thessaloniki, GR-54124 Thessaloniki, Greece; gkikae@auth.gr; 4 Department of Botany, School of Biology, Aristotle University of Thessaloniki, GR-54124 Thessaloniki, Greece; epanter@bio.auth.gr; 5 Laboratory of Pharmacology, Department of Pharmaceutical Sciences, Aristotle University of Thessaloniki, GR-54124 Thessaloniki, Greece; ivizir@pharm.auth.gr; 6 Department of Pharmacy, Faculty of Health and Medical Sciences, University of Copenhagen, DK-2100 Copenhagen, Denmark; anette.mullertz@sund.ku.dk; 7 Bioneer: FARMA, Faculty of Health and Medical Sciences, University of Copenhagen, DK-2100 Copenhagen, Denmark

**Keywords:** self-emulsifying drug delivery systems, amphotericin B, room temperature ionic liquids, Caco-2 cells, cytotoxicity

## Abstract

Amphotericin B (AmpB), one of the most commonly used agents in the treatment of severe fungal infections and life-threatening parasitic diseases such as visceral Leishmaniasis, has a negligible oral bioavailability, primarily due to a low solubility and permeability. To develop an oral formulation, medium chain triglycerides and nonionic surfactants in a self-nano-emulsifying drug delivery system (SNEDDS) containing AmpB were combined with room temperature ionic liquids (RTILs) of imidazolium. The presence of ionic liquids significantly enhanced the solubility of AmpB, exhibited a low toxicity and increased the transport of AmpB across Caco-2 cell monolayers. The combination of RTILs with a lipid formulation might be a promising strategy to improve the oral bioavailability of AmpB.

## 1. Introduction

Lipid-based formulations are emerging as an interesting formulation strategy for providing bioavailability-enhancing mechanisms for poorly water-soluble drugs belonging to Biopharmaceutics Classification System (BCS) Class II or IV [1,2]. The oral delivery of these drugs is affected by their low bioavailability and lack of dose solubility [3]. Another important factor that also affects the oral bioavailability of BCS IV drugs is poor intestinal permeability [3]. Hence, to enhance their solubility, lipid-based formulations have been successfully used as oral delivery systems [4]. Self-nano-emulsifying drug delivery systems (SNEDDS) are one of the most special and interesting lipid-based formulations [4] and are considered as a promising approach for improving the rate and extent of absorption of BCS II or IV drugs [5,6]. SNEDDS are defined as isotropic mixtures of oils, surfactants and co-solvents that have a unique ability of forming a fine oil in water nanoemulsions upon gentle agitation by dilution in an aqueous environment [7,8,9]. The droplet sizes of these nanoemulsions are between 20 and 200 nm [8,10]. SNEDDS induce the formation of oil droplets, which in turn may facilitate the transport of the active across the intestinal membrane [11].

**Figure 1:**
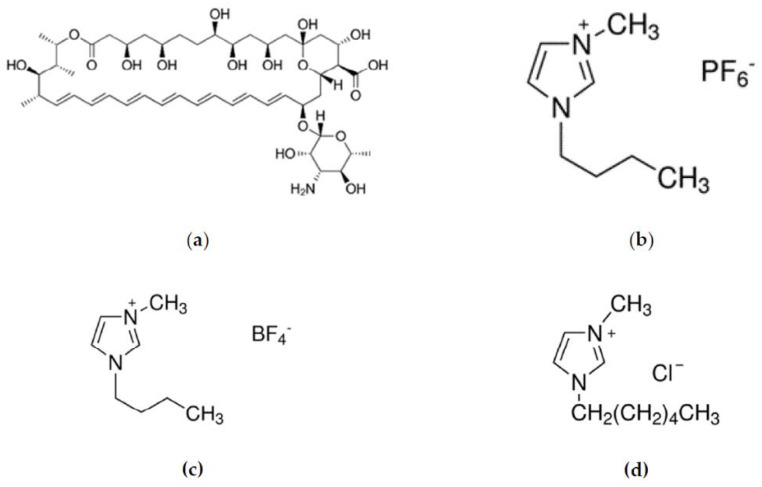
Structure of (**a**) AmpB, (**b**) BMIMPF_6_, (**c**) BMIMBF_4_ and (**d**) HMIMCl.

AmpB is a polyene antifungal antibiotic and belongs to BCS Class IV, which is characterized by a low permeability and low solubility [12]. This drug remains the most effective antifungal medication for severe fungal infections and visceral Leishmaniasis [13]. Worldwide, 12–15 million people are infected with visceral Leishmaniasis, and it is estimated that 70,000 deaths are caused every year [14]. AmpB, when administered orally, shows a poor gastrointestinal absorption and negligible bioavailability due to the hydrophobicity of its polyene structure (Figure 1a) [15]. However, it should remain solubilized in the gastrointestinal tract (GIT) to facilitate its absorption across the intestinal wall. In this context, an attempt was made to incorporate room temperature ionic liquids (RTILs), which are liquids at ambient temperature, into SNEDDS, as a means of increasing the solubility, which in turn might increase the absorption of the Active Pharmaceutical Ingredient (API) in the gastrointestinal tract [16,17,18,19]. RTILs are organic salts, and they are often called “designer solvents” since their physicochemical properties depend on cations and anions which can be varied. Furthermore, imidazolium or pyridinium ions can be used as organic cations of RTILs, combined with a variety of anions such as hexafluorophosphate or tetrafluoroborate (Figure 1) [18]. In addition, RTILs appear as promising candidates for enhancing the aqueous solubility of hydrophobic drugs via the selection of the adequate cation/anion combinations [20]. RTILs are hydrotropic compounds, which are capable of substantially increasing the solubility of hydrophobic substances in water. There are three main hypotheses that have been proposed in order to explain the hydrotropic solubilization. According to the first approach, a formation of a complex between the solute and the hydrotrope may occur. Furthermore, the hydrotropes may change the solvent structure around the solute and can be therefore considered as structure makers or breakers. Finally, the co-aggregation of the solute with the hydrotropes above a minimum hydrotrope concentration has been proposed as the most likely mechanism [21,22]. On the other hand, RTILs have generated considerable interest in fields as broad as catalysis, extraction, energy storage and CO_2_ capture, but also in skin delivery as permeation enhancers [23,24].

Owing to their properties in the current study, three different types of RTILs of imidazolium, namely 1-Butyl-3-methylimidazolium hexafluorophosphate (BMIMPF_6_) (Figure 1b), 1-Butyl-3-methylimidazolium tetrafluoroborate (BMIMBF_4_) (Figure 1c) and 1-Hexyl-3-methylimidazolium chloride (HMIMCl) (Figure 1d), were combined with a SNEDDS preconcentrate (SNEDDS:RTILs) containing AmpB and were further evaluated in vitro. The RTILs varied in alkyl moieties [bmim, butyl-] and [hmim, hexyl-], and in the type of anions PF_6_^−^, BF_4_^−^ and Cl^−^. Previous studies have shown that the type of ion and the length of the alkyl chain might affect the solubility of the APIs [16], whereas their toxicity is negligible in relation to the Caco-2 monolayers [16]. To this end, this was a two-tier study, with one aspect focusing on the investigation of imidazolium-based RTILs for the enhancement of the solubility of AmpB when co-formulated in SNEDDS, and another focusing on the cytocompatibility of the developed SNEDDS on the Caco-2 cells monolayers.

## 2. Materials and Methods

### 2.1. Materials

Amphotericin B was obtained from Alfa Aesar (Kandel, Germany). Propylene glycol, ionic liquids (namely 1-Butyl-3-methylimidazolium hexafluorophosphate (BMIMPF_6_) (M.W. 284.18 g/mol), 1-Butyl-3-methylimidazolium tetrafluoroborate (BMIMBF_4_) (M.W. 226.02 g/mol) and 1-Hexyl-3-methylimidazolium chloride (HMIMCl) (M.W. 202.72 g/mol)), Nile Red, bovine serum albumin Triton X-100, coumarin-6, formaldehyde and N-2-hydroxy-ethylpiperazine-N′-2-ethanesulfonic acid (HEPES) were obtained from Sigma-Aldrich (Steinheim, Germany). Dulbecco’s modified eagle’s medium (DMEM), penicillin streptomycin, fetal bovine serum (FBS) and Hanks’ Balanced Salt Solution (HBSS) were purchased from Gibco™ (Life Technologies, Grand Island, NY, USA). ProLong Gold Antifade Mountant with 4′,6-diamidino-2-phenylindole (DAPI) was purchased from Thermo Fischer Scientific (Walham, MA, USA). 3-(4,5-dimethylthiazol-2-yl)-5-(3-carboxymethoxyphenyl)-2-(4-sulfophenyl)-2H-tetrazolium / phenazine methosulfate (MTS/PMS) assay was purchased from Promega (Madison, WI, USA). Polyoxyl 40 hydrogenated castor oil (Kolliphor RH40) was kindly supplied by BASF (Ludwigshafen, Germany), and Captex^®^ 355 (triglycerides of caprylic/capric acid) was kindly supplied by ABITEC (Columbus, OH, USA). SIF powder for preparing simulated intestinal fluids was obtained from Biorelevant.com (London, UK). Distilled water was used in all experimental procedures. All other chemicals and solvents were of analytical grade and used as received unless otherwise specified.

### 2.2. Methods

#### 2.2.1. Preparation and Evaluation of SNEDDS Formulations

A previously developed SNEDDS formulation was prepared using Captex^®^ 355 as the oil vehicle and Kolliphor RH40 as the non-ionic surfactant [25]. Briefly, in a 1:1 blend of oil and surfactant, 10% *w/w* of Propylene Glycol was added as co-solvent. The mixture was stirred at 37 °C until homogeneity. Subsequently, three different RTILs were incorporated into the SNEDDS preconcentrates as follows: 0.3 g of IL was mixed with 0.7 g of SNEDDS), and they were stirred until complete homogenization (SNEDDS:BMIMBF_4_, SNEDDS:BMIMPF_6_ and SNEDDS:HMIMCl), forming clear isotropic preconcentrates.

The SNEDDS preconcentrates (in the presence and absence of RTILs) were evaluated by visual inspection over a period of 24 h at ambient temperature. The formulations were characterized as stable when no precipitation or phase separation occurred at the end of this time period. SNEDDS were dispersed in water, and a dilution factor of 100 (*v/v*) times was studied. Dispersions of all formulations were evaluated by Dynamic Light Scattering (DLS). In addition, the self-emulsification time was also assessed. The emulsification time, the time required by the preconcentrate to form a homogeneous nanoemulsion [26], was determined by the addition of the proper amount of SNEDDS preconcentrate to water (1:100 *v/v* dispersion ratio). The addition of SNEDDS was conducted by pipetting under gentle agitation. The self-emulsification time is the time required by the preconcentrate to form a homogeneous nanoemulsion when the disappearance of the SNEDDS preconcentrate is observed visually [26]. The average droplet diameter and the polydispersity index (PDI) of the dispersions were measured by DLS and ζ-potential measurements at 25 °C using a Malvern Nanosizer ZS (Malvern Instruments, Malvern, UK).

#### 2.2.2. Fluorescence Spectroscopy

The miscibility of the API with the preconcentrates was monitored using a lipophilic probe by means of fluorescence spectroscopy. The hydrophobic fluorescent marker Nile Red (NR) was used as a model drug, and the ability of oils to accommodate lipophilic compounds was elucidated because of solvatochromism of NR. SNEDDS preconcentrates were dispersed in water, at a 1:100 ratio, with a final concentration 26 μM of Nile Red [27]. A fluorescence spectroscopy (RF 5301, Shimadzu, Kyoto, Japan) was used to quantified the drug content. The fluorescent intensity of the samples was determined at excitation (EX) wavelengths of 546 nm. The emission (EM) spectra were recorded from 550 to 700 nm, and the excitation and emission slit widths were set at 1.5.

#### 2.2.3. Solubility Studies and Drug Loaded Formulations

To evaluate the effect of RTILs on the solubility of AmpB, an excess amount of the drug was added to the SNEDDS and SNEDDS:RTILs preconcentrates. After vortex mixing, the samples were kept under gentle agitation at ambient temperature for three days for equilibration (protected from light). The equilibrated samples were centrifuged at 10,000× *g* for 15 min, followed by filtration through 0.45-μM PVDF filters, and the supernatants were taken and diluted with MeOH-H_2_O (50:50). The amount of AmpB thus obtained was quantified by LC-MS/MS. For the AmpB-loaded SNEDDS, the results for the maximum amount of solubility were used for every formulation.

Solubility studies in distilled water were performed by mixing 50 μL of each preconcentrate with 4950 μL of water (100 times dilution) and vortexing to ensure that the formulation was well mixed. The dilutions were kept under gentle agitation at ambient temperature for 2 h. The samples were centrifuged at 10,000× *g* for 10 min, and the supernatants were then evaporated until dryness. The dry residue was reconstituted with 50 μL of MeOH-H_2_O (50:50), and the samples were analyzed by LC-MS/MS.

#### 2.2.4. LC-MS/MS Analysis

Chromatography separation was achieved using an Alliance HT Waters 2790 combined with a SCIEX API 3200TM mass spectrometer in positive mode (+ ESI). Separation was held with an XTerra MS C18 (2.1 mm × 50 mm, 3.5 μM) column (Waters, Dublin, Ireland) and mobile phases A: water (0.04% acetic acid, pH = 3.5) and B: methanol. Gradient elution was started from 60% B and increased to 100% B in 4 min. The flow rate of the mobile phase and the column oven temperature were set at 0.4 mL/min and 40 °C, respectively. Multiple Reaction Monitoring was performed to achieve MS/MS transitions of AmpB: *m/z* 924.3 (parent ion) to 743.3 (daughter ion) as the quantifier ion and *m/z* 924.3 (parent ion) to 267.2 (daughter ion) as the qualifier ion. The declustering potential and collision energy were applied at 51 V and 35 V, respectively. The source temperature was fixed at 450 °C, and the spray voltage was set at 4500 V. Analyst Software was used for data acquisition and analysis. The calibration standards of AmpB were diluted in MeOH–H_2_O (1:1 *v/v*), and the calibration curve was linear (*R*^2^ = 0.999) at a concentration range of 2 ng/mL to 2 μg/mL.

#### 2.2.5. Stability of Formulations

A long-term stability study was performed by storing the SNEDDS preconcentrates at room temperature. At two- and four-week intervals, samples were taken, and the size of the droplet formed upon dispersion (1:100 ratio) was determined. The chemical stability of AmpB in the SNEDDS preconcentrates was also evaluated over time. The degradation and/or precipitation of freshly prepared AmpB-loaded SNEDDS was evaluated upon storage over time. Moreover, the stability of AmpB in simulated gastric fluid (SGF) at pH 1.2 (2.0 g NaCl, 80 mL 1 M HCl/L) and intestinal fluids was evaluated. A mixture of sodium taurocholate and egg lecithin (SIF powder) was used for the preparation of fasted and fed intestinal simulated fluids (FaSSIF pH 6.5 and FeSSIF pH 5). The SNEDDS were incubated in SGF or in SIF at a 1:100 (*v/v*) dilution at 37 °C with vigorous stirring. The incubation times were 10, 30 and 120 min. The concentration of AmpB was determined at each time point after complete solubilization in MeOH:H_2_O 50:50 *v/v* to clarify the samples. The amount of drug for each study was analyzed by LC-MS/MS.

#### 2.2.6. Transport of AmpB across Caco-2 Monolayers

For the in vitro testing of oral formulations, a useful surrogate for human intestinal epithelium is provided by Caco-2 cells. The cells were cultured in DMEM supplemented with 584 g/mL L-glutamine, 90 U/mL penicillin, 90 g/mL streptomycin, 1% (*w/v*) nonessential amino acids and 10% (*v/v*) fetal bovine serum. Cells were maintained at 37 °C in a humidified atmosphere of 5% CO_2_/95% (*v/v*) air and 90% relative humidity. Exponentially growing cells were used for all studies.

Caco-2 cells were plated on 12-well Transwell inserts (Corning; Sigma-Aldrich, Steinheim, Germany) with a porous polycarbonate membrane of 0.4 μM pore size and 1.13 cm^2^ growth area. The density of the cells was 2 × 10^5^ cells/cm^2^, and passage numbers 14–15 were used. The cells were grown for three weeks, and the medium was changed every other day. After this period, the transport study was started. In brief, the monolayers were washed with HBSS buffer at 37 °C. The inserts were then immersed in transport buffer, 1000 μL on the basolateral side and 500 μL of diluted SNEDDS (1:100 *v/v*) on the apical side. At 120 min, 100 μL from the basolateral side were collected. After centrifugation at 10,000× *g* for 10 min, the clear supernatants of the samples were evaporated until dryness. Reconstitution of the dry samples was performed with 50 μL of MeOH–H_2_O (50:50), and then the samples were analyzed by LC-MS/MS.

#### 2.2.7. Measurement of Transepithelial Electrical Resistance

The effect of the SNEDDS formulations on the Caco-2 cell monolayer integrity was evaluated by monitoring the transepithelial electrical resistance (TEER) as a function of time, using a Voltohmmeter EVOM (World Precision Instruments, Inc., Sarasota, FL, USA). Monolayers displaying TEER values above 500 Ω cm^2^ were considered appropriate for starting the experiment. 2, 1 and 0.5 h before the transport study, at the beginning (0 h) and at the end (2 h), the TEER values were recorded. After the end of the experiment, the formulations were removed and the monolayers were washed with HBSS buffer. Fresh growth medium was added, and the TEER values were recorded for an additional 1320 min to assess the ability of the cells to recover from treatment.

#### 2.2.8. Viability Studies

The biocompatibility of SNEDDS was assessed by performing the MTS/PMS assay. Caco-2 cells diluted in culture medium at a density of 6 × 10^4^ cells/cm^2^ were seeded onto 96-well plates and were then maintained for 48 h to attach. Prior to treatment, the culture media was removed and replaced by HBSS buffer for washing. The cells were incubated with the SNEDDS dispersed in HBSS at a ratio of 1:100, at 37 °C for 1, 2 and 4 h. Subsequently, the formulations were removed, the cells were washed with buffer and 100 μL of colorimetric MTS/PMS assay was added to each well for 1–2 h at 37 °C (90 rpm). The assay is based on the reduction of MTS tetrazolium by mitochondria in viable cells to a colored formazan product that is soluble in tissue culture medium. The well plate was protected from light. The generated formazan dye was measured by absorbance at 492 nm in a microplate reader (Multiskan MS photometer type 352; Labsystems, Helsinki, Finland).

Cell viability was expressed as the percentage of absorbance of the sample relative to control, according to the following Equation:

(1) $$\mathrm{Viability}\;\left(\%\right)=100\times \frac{A-P}{N-P}$$

where A, P and N are the absorbance obtained for the tested formulations, positive control (cells treated with 1% Triton X-100) and negative control (cells treated with HBSS only), respectively.

#### 2.2.9. Cellular Uptake Studies

Uptake studies of coumarin-6 were conducted by Confocal Laser Scanning Microscopy (CLSM). Caco-2 cells from passage numbers 38–41 were seeded on 6-well plates, and a sterilized coverslip was placed at the bottom of each well. The starting concentration of cells was 4.5 × 10^4^ cells/cm^2^, and they were cultured for five days to form a monolayer before the initiation of the uptake experiment. The cells were then incubated in the presence of either formulations (1:100-diluted SNEDDS with 0.5 μg/mL coumarin-6) or coumarin-6 solution in HBSS as control. After 2 h, the supernatant was discarded, and the attached cells were washed three times with HBSS and were subsequently treated with a fixation solution of 4% formaldehyde for 20 min. The cells were washed with HBSS buffer and permeabilized with 0.3% Triton X-100 for 15 min. Cell nuclei were stained with Prolong Gold Antifade Reagent with DAPI and left in the dark at 4 °C. Images was acquired by Confocal Laser Scanning Microscopy (Zeiss LSM780 CLSM, Oberkochen, Germany) using the appropriate filters. A 40× oil immersion lens was used.

#### 2.2.10. Statistical and Data Analysis

All data were presented as mean values of four repetitions and the standard deviation (± S.D.). The results were compared via the Student’s t-test. A p-value of less than 0.05 was considered as being statistically significant.

## 3. Results and Discussion

### 3.1. Characterization of SNEDDS

The SNEDDS formed a fine nanoemulsion over 38–52 s upon dispersion in water under mild agitation. All SNEDDS preconcentrates were visually inspected and did not show phase separation over a period of 24 h.

**Figure 2:**
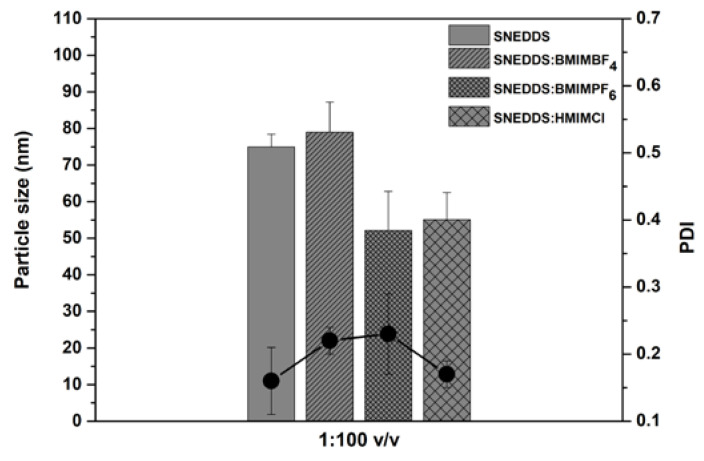
The droplet size (nm) and polydispersity index (PDI) of SNEDDS (without AmpB) dispersed in water at a 1:100 *v/v* dilution.

The droplet size and polydispersity index (PDI) of SNEDDS dispersed in water at 1:100 *v/v* dilution are shown in Figure 2. The droplet size of SNEDDS in water was 75 ± 3.4 nm, whereas for SNEDDS:BMIMBF_4_, SNΕDDS:BMIMPF_6_ and SNEDDS:HMIMCl they were 79 ± 8 (*p* > 0.05), 52 ± 11 (*p* < 0.05) and 55 ± 7 nm (*p* < 0.05), respectively. It has been reported that the addition of imidazolium cations to the formulation can change the arrangement of the non-anionic and ionic excipients on the surface of the oil droplets. Thus, this addition can lead to smaller droplet sizes and greater long-term stability [23]. The PDI values were less than 0.5 for all formulations. The ζ-potential was −5.3 ± 0.4 mV for SNEDDS and −2.3 ± 0.9, −14.1 ± 2.3 and −1.8 ± 0.6 mV for SNEDDS:BMIMBF_4_, SNEDDS:BMIMPF_6_ and SNEDDS:HMIMCl, respectively.

### 3.2. Fluorescence Spectroscopy

The fluorescence spectra of SNEDDS (Supporting Figure S1) were recorded, and the solvatochromic behavior of NR was monitored. In all cases with RTILs, it was clear that the samples exhibited a lower fluorescence intensity compared to the SNEDDS. The reduced intensity of fluorescence seen for RTIL-free droplet samples relative to that seen for their RTIL-loaded congeners is consistent with the reduced capacity of the latter to accommodate NR. The fluorescence emission intensities from NR in SNEDDS:HMIMCl were lower than those from NR in SNEDDS:BMIMBF_4_ and SNEDDS:BMIMPF_6_ respectively, indicating a greater exposure to the aqueous phase. Thus, the fluorescent intensity is proportionally related to the size of the particles hosting the fluorescent dye [27,28].

### 3.3. Solubility Studies

**Figure 3:**
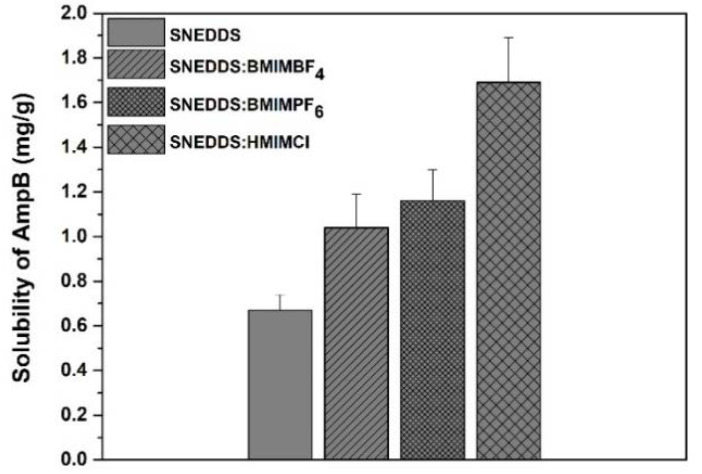
Solubility of AmpB in SNEDDS and SNEDDS:RTILs.

The solubility of AmpB was 0.67 ± 0.07 mg/g in the SNEDDS, whereas the presence of RTILs increased the solubility of AmpB as follows: 1.04 ± 0.15 mg/g for SNEDDS:BMIMBF_4_, 1.16 ± 0.14 mg/g for SNEDDS:BMIMPF_6_ and 1.69 ± 0.2 mg/g for SNEDDS:HMIMCl, as shown in Figure 3. Significantly higher solubilities were obtained when replacing 33% of SNEDDS with RTILs (1.55-fold for SNEDDS:BMIMBF_4_, 1.73-fold for SNEDDS:BMIMPF_6_ and 2.52-fold for SNEDDS:HMIMCl respectively) (*p* < 0.05). This might be attributed to the fact that the PF_6_ salts are more hydrophobic than the BF_4_ salts [24] and therefore are expected to dissolve AmpB better. In addition, the high solubility of AmpB with HMIMCl might be attributed to the longer alkyl chain (hexyl- versus butyl-for the other two) in the imidazolium cation. The results are in broad agreement with previous studies using imidazolium salts (PF_6_^−^,BF_4_^−^,Cl^−^) as solvents for poorly water-soluble model drugs [18].

The solubilities of the tested formulations containing AmpB in water were 1170 ± 69 ng/mL for the plain SNEDDS formulation, 1470 ± 186 ng/mL for SNEDDS:BMIMBF_4_, 1610 ± 112 ng/mL for SNEDDS:BMIMPF_6_ and 1810 ± 77 ng/mL for SNEDDS:HMIMCl, respectively. As evidenced in Table 1, the incorporation of AmpB in the SNEDDS increased the droplet size and PDI of all formulations. The droplet size of AmpB-containing SNEDDS was significantly higher (*p* < 0.05) compared to its empty congener (an increase from 75 to 154 nm). When RTILs and AmpB were incorporated into the SNEDDS preconcentrates, the size of the formed droplets ranged between 118 and 166 nm, which is significantly higher than for the corresponding drug-free SNEDDS (52 to 79 nm, *p* < 0.05). The lower particle size recorded for SNEDDS:BMIMPF_6_ and SNEDDS:HMIMCl, when compared to the empty SNEDDS formulations, might be explained in terms of membrane fluidity, as it appears that these RTILs might increase the fluidity of the oil core of the droplets, allowing NR (Supporting Figure S1) to penetrate deeper (as evidenced by the fluorescence emission spectra) and allowing for a better packing of the carrier. In the case of SNEDDS:BMIMBF_4_, the larger particle size of the produced droplets might be attributed to the less hydrophobic nature of BF_4_ when compared to PF_6_ [29], which in turn affects its packing. The presence of the ionic liquids slightly decreased the surface charge of the SNEDDS formulation (with the exception of BMIMPF_6_) in absolute values, and this might be correlated with the ionic character of these liquids, as they can act as cationic surfactants [30]. Upon addition of AmpB, the ζ-potential of the emulsion droplets increased (Table 1). Although the ζ-potential values of all the tested dispersions was relatively low, droplet stability might be achieved by steric hindrance due to the presence of Kolliphor RH40.

### 3.4. Stability of Formulations

**Figure 4:**
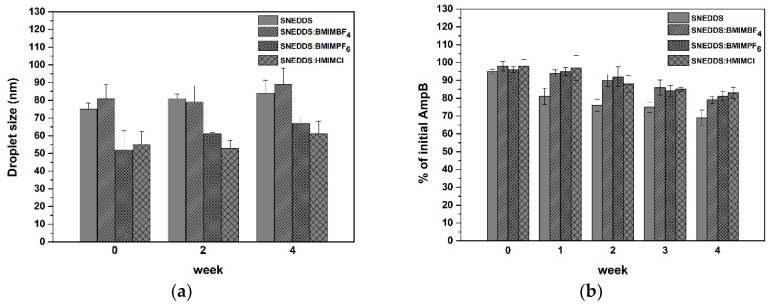
Stability of (**a**) empty and (**b**) drug-loaded formulations upon dilution in water (1:100) over a period of four weeks.

The size of the droplets formed after dispersion of the SNEDDS at 0, 2 and 4 weeks after production is depicted in Figure 4a. No significant difference in the droplet size distribution was observed for any of the SNEDDS formulations. This is in accordance with a previous report, according to which RTILs can keep the formulation stable over time, as a result of which the droplet size distributions of the formed emulsions remain constant during storage [23].

The AmpB in all SNEDDS over a period of four weeks showed a gradual reduction, as evidenced by Figure 4b. The SNEDDS recorded the highest loss of drug (up to 31% loss), while the SNEDDS:HMIMCl recorded a lower decrease of drug (ca. 17% loss) (*p* < 0.05). The decrease in the AmpB concentration was due to drug precipitation, which was visually observed. LC-MS/MS chromatograms indicating the absence of degradation of AmpB are presented in Supporting Figure S2.

**Figure 5:**
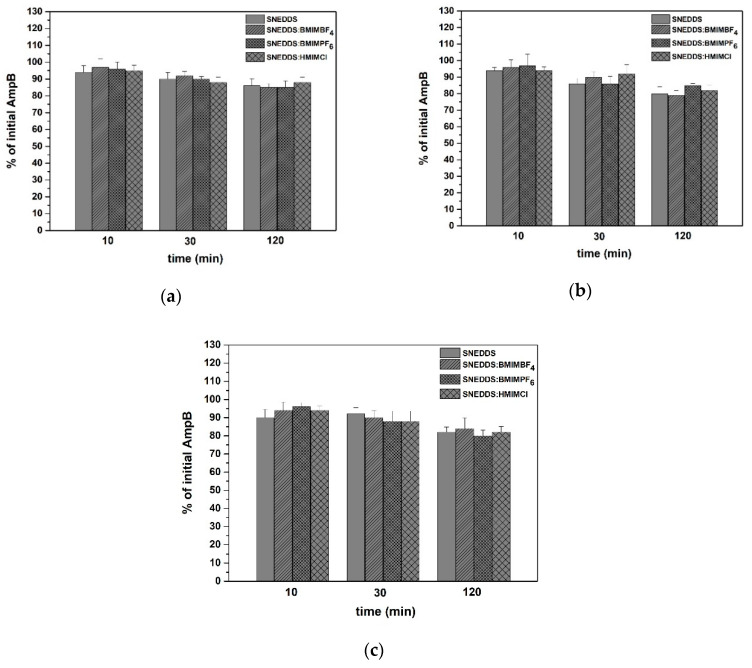
Stability of AmpB in SNEDDS dispersed in (**a**) SGF, (**b**) FaSSIF and (**c**) FeSSIF over time.

The stability of AmpB in the four SNEDDS dispersed in simulated gastric and intestinal fluids was also investigated. In all SNEDDS, the AmpB exhibited a sufficient stability after dispersion in SGF (Figure 5a), FaSSIF (Figure 5b) and FeSSIF (Figure 5c). There was a reduction of the initial amount of AmpB over time, not lower than 80% in all cases (*p* < 0.05). The solubility of AmpB in SGF, FaSSiF and FeSSiF media was 77.0 ± 0.53 μg/mL, 18.2 ± 0.33 μg/mL and 30.7 ± 0.4 μg/mL, respectively. In the case of plain SNEDDS, SNEDDS:BMIMBF_4_, SNEDDS:BMIMPF_6_ and SNEDDS:HMIMCl, the final concentrations of AmpB were 6.7 μg/mL, 10.2 μg/mL, 11.6 μg/mL and 16.9 μg/mL, showing that the API was below the non-crystalline state in all of the tested media.

### 3.5. Transport Studies across Caco-2 Monolayers

**Figure 6:**
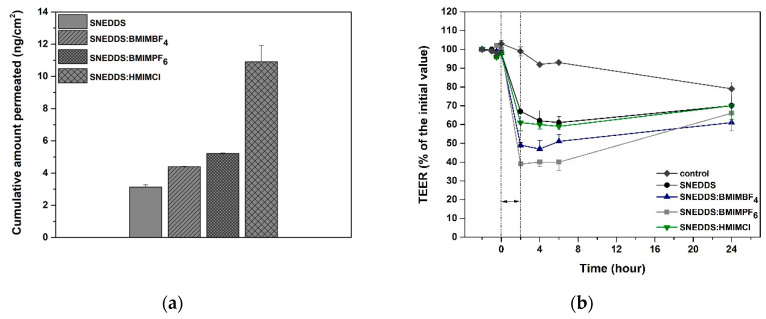
(**a**) Total accumulated amount of AmpB across the Caco-2 monolayer and (**b**) TEER values.

The transports of AmpB from SNEDDS and SNEDDS:RTILs, expressed as the cumulative amounts permeated at 2 h, are shown in Figure 6a. The cumulative amounts of AmpB transported across the Caco-2 monolayer over 2 h were 4.39 ± 0.03 ng/cm^2^, 5.22 ± 0.03 ng/cm^2^ and 10.91 ± 1.01 ng/cm^2^ for SNEDDS:BMIMBF_4_, SNEDDS:BMIMPF_6_ and SNEDDS:HMIMCl, respectively, which was higher than for the SNEDDS (3.13 ± 0.15 ng/cm^2^). The incorporation of BMIMBF_4_ and BMIMPF_6_ into SNEDDS did not show a significant increase in the amount of AmpB when compared to the control (*p* > 0.05). On the contrary, SNEDDS:HMIMCl improved the transport of AmpB when compared to SNEDDS (*p* < 0.05). This might be attributed to the longer alkyl chain in the imidazolium cation, which further increased the solvency of AmpB and could in turn affect the transport rate of the API across the Caco-2 monolayers.

In general, medium chain triglycerides, such as Captex^®^355, are known to possess permeation-enhancing properties by opening tight junctions [31,32,33]. The absorption enhancement has been related to a redistribution of the cytoskeleton and structural dilatations in the tight junctions [34]. On the other hand, ionic liquids can also improve drug absorption with their positive effect on permeation attributed to their ionic character. It has been previously reported that imidazolium-based RTILs can fluidize the cell membrane in order to create pathways for the diffusion of molecules [35].

The incubation of the Caco-cell monolayers with SNEDDS caused a reduction of TEER values of up to 60% at 2 h (Figure 6b) when compared to no decrease (99%) for the control (*p* < 0.05). This reduction might be attributed to the ester derivatives of C8 and C10 fatty acids of Captex^®^ 355, due to their ability to open the tight junction [36,37]. Upon removal of SNEDDS and substitution of the apical medium with fresh growth medium, the monolayers started to slowly recover, and a constant increase in resistance was recorded. More specifically, at 24 h the TEER reached 70% of the initial TEER for SNEDDS and SNEDDS:HMIMCl, 66% for SNEDDS:BMIMPF_6_ and 61% for SNEDDS:BMIMBF_4_, when compared to 79% for the control (*p* < 0.05).

Moreover, the solubility studies revealed that the presence of RTILs significantly increased the solubility of AmpB when compared to the plain SNEDDS formulation. These data show that the increased transport of AmpB across Caco-2 monolayers in the presence of RTILs might be attributed to the ability of RTILs to both increase the solubility of AmpB and, at the same time, cause a reduction of the TEER values.

### 3.6. Cell Viability

**Figure 7:**
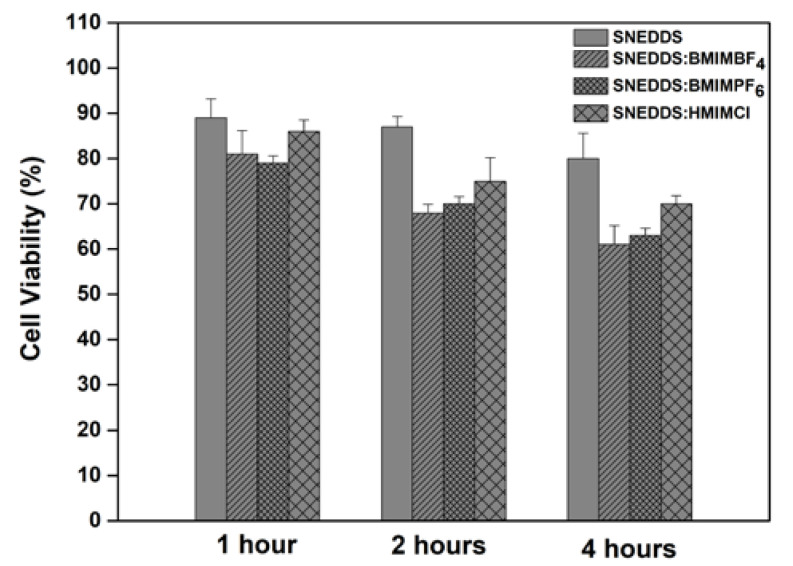
Caco-2 cell viability after exposure of cultures to SNEDDS for 1 h, 2 h and 4 h.

The cytocompatibility of the different SNEDDS was assessed on the Caco-2 cell line in a time-dependent manner using the MTS/PMS assay (Figure 7). No obvious cytotoxicity (> 80%) was observed for the cell treatment with SNEDDS at the different time points that were measured (1, 2 and 4 h). Cell viability was retained at over 61% for all SNEDDS with RTILs, showing a decrease as a function of time (*p* < 0.05). Approximately 61%, 63% and 72% of the Caco-2 cells were viable after 4 h of the experiment for BMIMBF_4_, BMIMPF_6_ and HMIMCl, respectively. These values indicate that SNEDDS with RTILs exhibited a slightly toxic effect on the cells. The results obtained in the current study are in broad agreement with previous studies, where a low or moderate toxicity of RTILs has been observed in different cell lines [17,23,38].

### 3.7. Cellular Uptake

**Figure 8:**
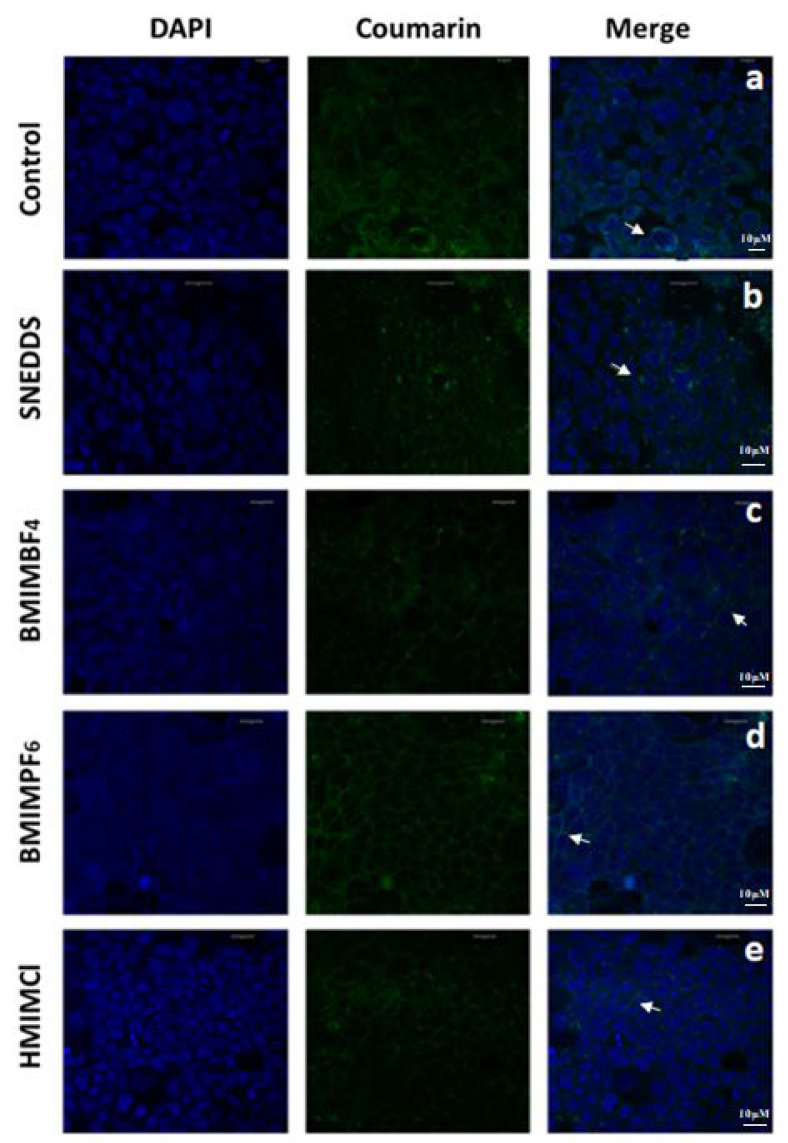
CLSM images of Caco-2 cells after 2 h of incubation of (**a**) coumarin-6 solution (control), (**b**) SNEDDS, (**c**) SNEDDS:BMIMBF_4_, (**d**) SNEDDS:BMIMPF_6_ and (**e**) SNEDDS:HMIMCl with the fluorescent coumarin-6 (green). DAPI is a blue-fluorescent DNA agent that allows the staining and visualization of nuclei. Total Scale bar: 10 μM. White arrows indicate the distribution of coumarin-6 in the cytoplasm and the intercellular spaces.

The cellular uptake and intracellular distribution of coumarin-6 in Caco-2 cells was investigated by CLSM after cell exposure to SNEDDS and SNEDDS:RTILs. The white arrow in Figure 8a shows that, upon incubation with Caco-2 monolayers, the coumarin-6 solution (control) is located close to the cytoplasm [39,40]. Images of SNEDDS containing coumarin-6, shown as green spots closely located to the nuclei and illustrated with a white arrow, are shown in Figure 8b. For SNEDDS containing BMIMBF_4_, the images show a more intense fluorescence of coumarin-6 in the intercellular spaces co-existing with green particles (Figure 8c) when compared to their plain congener, and this more intense fluorescence is shown by a white arrow. When BMIMPF_6_ is co-formulated with SNEDDS, the coumarin-6 fluorescence is also present in the intercellular space (Figure 8d). The fluorescence staining for BMIMPF_6_ formulations appears to be more intense when compared to BMIMBF_4_. Finally, when SNEDDS containing HMIMCl were incubated with Caco-2 monolayers, increased numbers of green particles and increased fluorescence staining were observed (Figure 8e). The area with increased green particles and fluorescence staining is shown by a white arrow. In parallel, this exposure of Caco-2 cells to SNEDDS and SNEDDS:RTILS does not appear to affect nuclei, since no substantial cell nuclei swelling and fragmentation was observed in the treated cultures when compared to the control.

## 4. Conclusions

Incorporating RTILs into SNEDDS formulations slightly increased the droplet size and the PDI, while decreasing the ζ-potential. The rank order of solubility of AmpB in SNEDDS was as follows: SNEDDS:HMIMCl > SNEDDS:BMIMPF_6_ > SNEDDS:BMIMBF_4_ > SNEDDS. The ability of RTILs to increase solubility depends on their anions (PF_6_^−^, BF_4_^−^, Cl^−^) and the length of their alkyl chain. Although promising, the solubility values obtained in the current study for AmpB in the presence of RTILs are relatively low from a clinical perspective. From this point of view, the possibility of co-formulating a second RTIL might be considered as a strategy to increase the drug’s solubility [16]. RTILs also enhanced the transport of AmpB across Caco-2 monolayers, with the same order as for the solubility results. In addition, the presence of RTILs resulted in more stably dispersed formulations when compared with their empty congeners, as evidenced by their droplet size distributions after four weeks. However, SNEDDS with RTILs exhibited a slightly toxic effect on the cells as a function of time. In conclusion, the array of new RTIL-lipid-based drug delivery strategies provide interesting perspectives, including a high drug-loading capacity and the ability to increase drug permeation profiles.

## Figures and Tables

**Table 1:** Droplet diameter (nm), polydispersity index (PDI) and ζ-potential (mV) in water at a 1:100 dilution with AmpB.

Sample	Size (nm)		PDI		ζ-Potential (mV)	
	without AmpB	with AmpB	without AmpB	with AmpB	without AmpB	with AmpB
SNEDDS	75 ± 3.4	154 ± 1	0.16 ± 0.05	0.35 ± 0.05	−5.3 ± 0.4	−10.6 ± 1.1
SNEDDS:BMIMBF_4_	79 ± 8	118 ± 9	0.22 ± 0.02	0.36 ± 0.04	−2.3 ± 0.9	−5.5 ± 0.2
SNEDDS:BMIMPF_6_	52 ± 11	166 ± 10	0.23 ± 0.06	0.33 ± 0.06	−14.1 ± 2.3	−8.9 ± 1.3
SNEDDS:HMIMCl	55 ± 7	150 ± 7	0.17 ± 0.02	0.27 ± 0.01	−1.8 ± 0.6	−3.8 ± 0.6

## Supplementary Materials

The following are available online at https://www.mdpi.com/1999-4923/12/8/699/s1, Figure S1: Fluorescence spectra of the SNEDDS diluted at 1:100 in water. Figure S2: Chromatograms of LC-MS/MS of (a) SNEDDS, (b) SNEDDS:BMIMBF4, (c) SNΕDDS:BMIMPF6 and (d) SNEDDS:HMIMCl after four weeks.
