# Supplementary material for: In Vitro Evaluation of Self-Nano-Emulsifying Drug Delivery Systems (SNEDDS) Containing Room Temperature Ionic Liquids (RTILs) for the Oral Delivery of Amphotericin B
Source: Pharmaceutics. 2020 Jul 25;12(8):699. doi: 10.3390/pharmaceutics12080699 (PMC7463809; doi:10.3390/pharmaceutics12080699)
Supplement: Supplementary file 1 [file pharmaceutics-12-00699-s001.pdf]

pharmaceutics

Supplementary Materials: In Vitro Evaluation of Self-Nano-Emulsifying Drug Delivery Systems (SNEDDS) Containing Room Temperature Ionic Liquids (RTILs) for the Oral Delivery of Amphotericin B

Eleni Kontogiannidou, Thomas Meikopoulos, Helen Gika, Emmanuel Panteris, Ioannis S. Vizirianakis, Anette Müllertz and Dimitrios G. Fatouros *

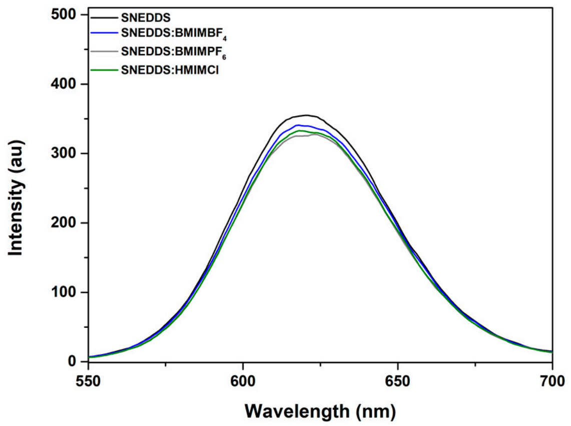

Figure S1. Fluorescence spectra of the SNEDDS diluted 1:100 in water.

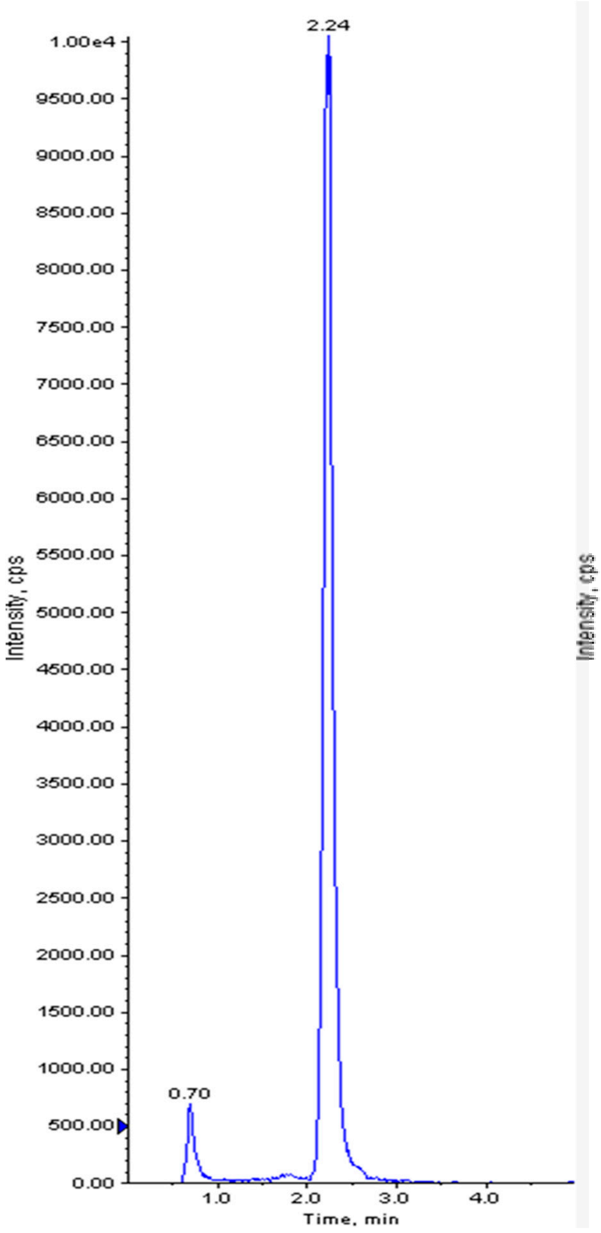
2.24
1.00e4
9500.00
9000.00
8500.00
8000.00
7500.00
7000.00
6500.00
6000.00
5500.00
5000.00
4500.00
4000.00
3500.00
3000.00
2500.00
2000.00
1500.00
1000.00
500.00
0.00
0.70
Intensity, cps
1.0
2.0
3.0
4.0
Time, min

(a)

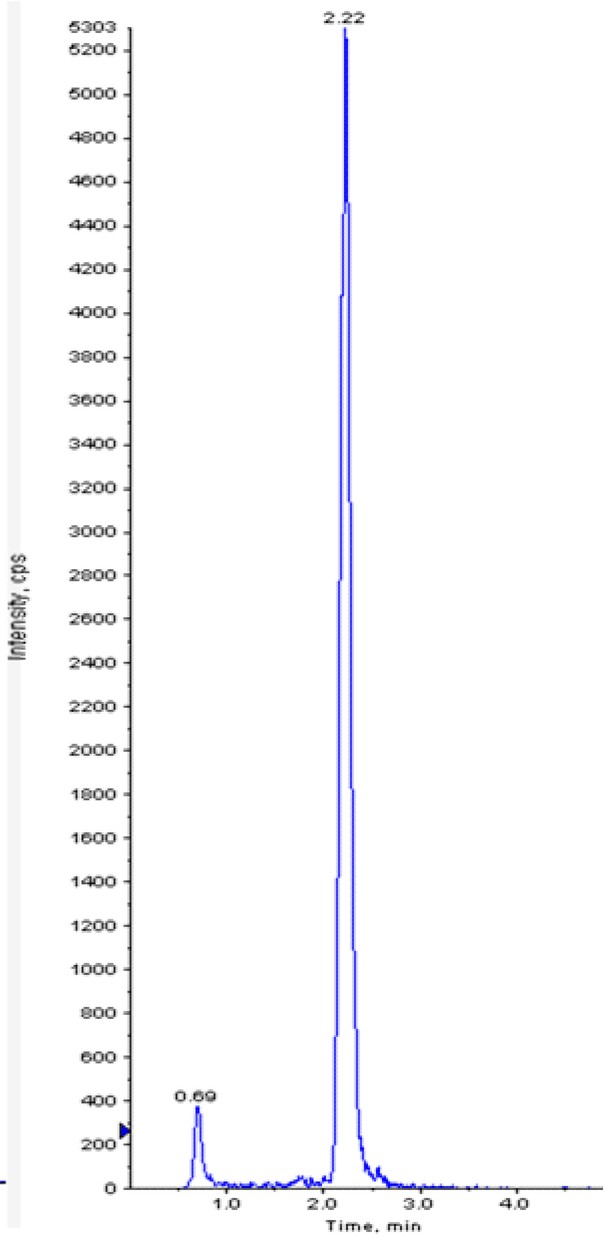
2.22
5303
5200
5000
4800
4600
4400
4200
4000
3800
3600
3400
3200
3000
2800
2600
2400
2200
2000
1800
1600
1400
1200
1000
800
600
400
200
0
0.69
Intensity, cps
1.0
2.0
3.0
4.0
Time, min

(b)

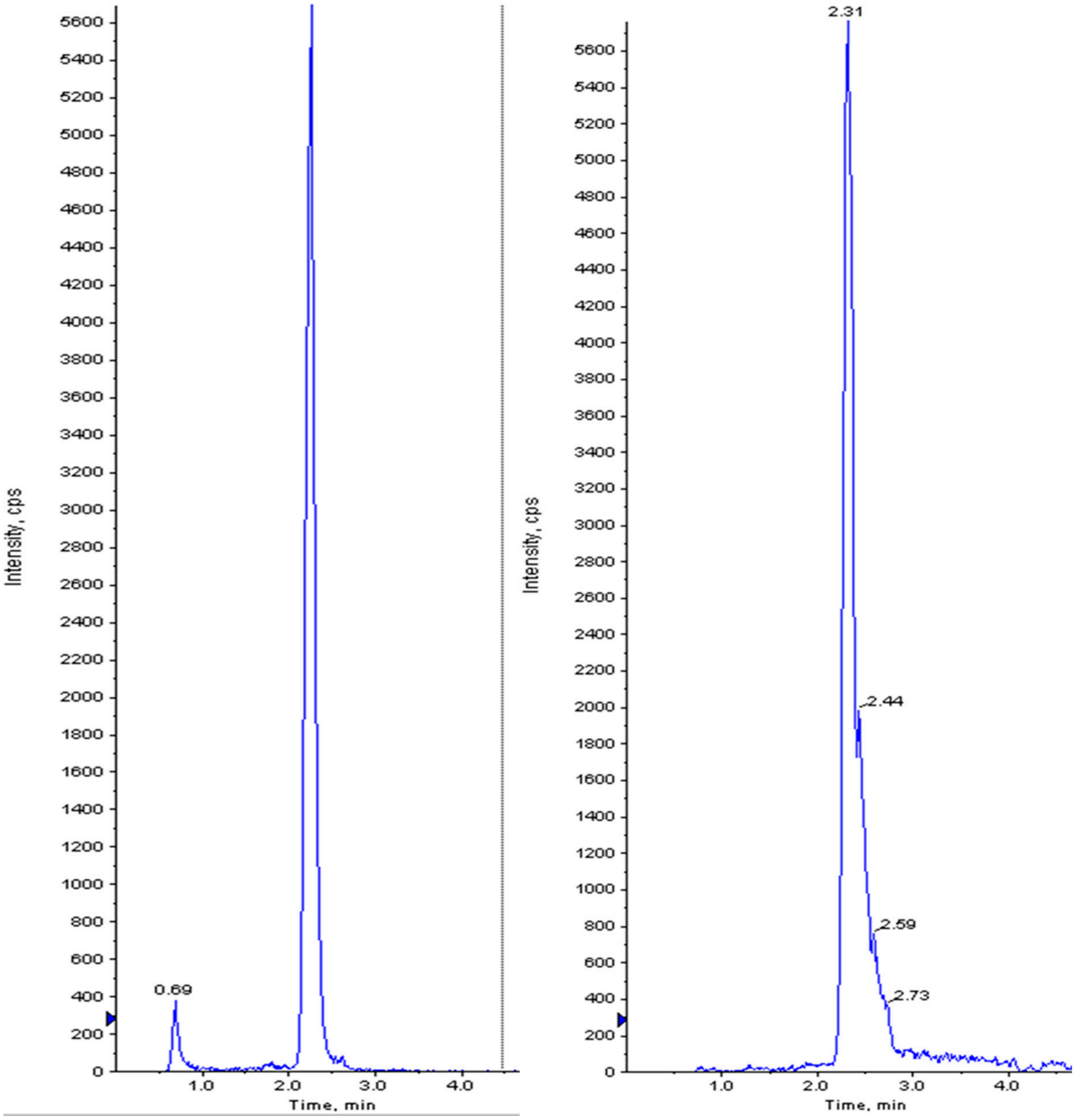

(c) (d)

Figure S2. Chromatograms of LC-MS/MS of SNEDDS (**a**), SNEDDS:BMIMBF$_4$ (**b**), SNEDDS:BMIMPF$_6$ (**c**) and SNEDDS:HMIMCl (**d**), after 4 weeks.
